# What Adult Electrocardiogram (ECG) Diagnoses or Findings are Most Important for Advanced Care Paramedics to Know?

**DOI:** 10.7759/cureus.16260

**Published:** 2021-07-08

**Authors:** Aaron Sibley, Mathew H MacLeod, Catherine Patocka, Jenny Yu, Henrik Stryhn, Trevor Jain

**Affiliations:** 1 Emergency Medicine, University of Prince Edward Island, Charlottetown, CAN; 2 Emergency Medicine, Holland College, Charlottetown, CAN; 3 Emergency Medicine, Cumming School of Medicine, University of Calgary, Calgary, CAN; 4 Department of Health Management, Atlantic Veterinary College, University of Prince Edward Island, Charlottetown, CAN

**Keywords:** electrocardiogram (ecg/ekg), prehospital medicine, medical education & training, delphi method, emergency medicine

## Abstract

Introduction: The interpretation of electrocardiograms (ECGs) is an essential competency in modern paramedicine. Although educational guidelines for paramedic ECG interpretation exist, they are broad, not evidence-based, and lack prioritization in a prehospital clinical context. We conducted this study to gain consensus among stakeholders (EMS physicians, paramedic educators, and paramedic clinicians) regarding which ECG diagnoses or findings are most important for a practising advanced care paramedic to know.

Methods: This study was an internet-based Delphi survey. We purposefully sampled participants in pairs (physician/paramedic) from all 10 Canadian provinces. Individuals rated a previously developed comprehensive list of emergency ECG diagnoses or findings on the importance of paramedic recognition and impact on prehospital care using a 4-point Likert scale. The consensus was achieved with a minimum of 75% agreement on Likert rating for a single diagnosis or finding during survey rounds one to three. When consensus was not reached, stability was defined as a shift of individual ratings between rounds of 20% or less.

Results: All 20 participants completed the first and second rounds of the survey, and 17 (85%) completed three rounds. Overall, 32 (26.4%) of 121 potentially important ECG diagnoses or findings reached consensus, 2 (1.7%) reached stability and 87 (71.9%) reached neither consensus nor stability. Twenty-one (17.4%) diagnoses or findings were considered “Very Important”, six (4.9%) “Important”, and five (4.1%) “Minimally Important”. In the first round of the survey, the mean rating of the importance of a paramedic knowing a specific ECG diagnosis or finding was lower in the physician group than the paramedic group on 85 (72%) of 118 initial diagnoses or findings.

Conclusion: We have created a list of ECG diagnoses or findings prioritized for the prehospital context that may assist paramedic educators in focusing on educational interventions. Many ECG diagnoses or findings failed to reach consensus or stability, demonstrating potential disagreement regarding clinical expectations for ECG knowledge among paramedics or physicians.

## Introduction

The interpretation of electrocardiograms (ECGs) is an essential skill in modern paramedicine. Although educational guidelines for paramedic ECG interpretation exist [1,2], they are broad, not evidence-based, and lack prioritization specifically in a prehospital clinical context.

In Canada, the National Occupational Competency Profile (NOCP) largely dictates the scope and practice of paramedics [1]. This document, produced by the Paramedic Association of Canada, helps to define the profession and promote consistency in paramedicine training and practice at a national level. According to the NOCP, advanced care providers, including Advanced Care Paramedics (ACP) and Critical Care Paramedics, must have proficiency in acquiring and interpreting 12 lead (and additional lead) ECGs while possessing skills such as manual defibrillation, electrical cardioversion, and transcutaneous pacing. Unfortunately, the document does not provide specific direction on what ECGs an advanced paramedic must be able to recognize.

In the United States, to be accredited by The Commission on Accreditation of Allied Health Education Programs, educational programs for Emergency Medical Services Professions must demonstrate that the curriculum offered meets or exceeds the content and competency of the latest edition of the National EMS Education Standards [3]. These instructional guidelines, published by the National Highway Traffic Safety Administration, provide an extensive list of ECG diagnoses and findings; however, they do not place value on the importance of knowing one diagnosis over another and have not been updated since 2009 [2]. 

A prioritized list of “must recognize” diagnoses or findings would assist educational institutions in focusing their ECG teaching and developing ECG interpretation competencies in both countries. We conducted this study to gain consensus among prehospital healthcare stakeholders (including paramedic clinicians, paramedic educators and Emergency Medical Services (EMS) physicians), what ECG diagnoses or findings are most important for a practising ACP to know.

This article was presented as a meeting abstract at the 2020 NAEMSP Annual Scientific Meeting on January 10, 2020.

## Materials and methods

Ethics

The Holland College Research Ethics Board, Charlottetown, PE, approved this study.

Design/Participants

This study was an internet-based Delphi survey with a predetermined maximum of three rounds. We purposively sampled [4] participants in pairs (EMS physician, Advanced Care Paramedic) from all ten Canadian provinces to participate as content experts. We invited participants based on their pre-existing relationships with the primary author through work on national committees and prior research collaborations. When a prior relationship was not present in a specific province, we used a snowball technique [4] to approach participants. Because prehospital guidelines and protocols may be specific to individual provinces, regions and EMS services, we sought representation from each province to provide a national perspective on current paramedic practice. The participants are all recognized as leaders in EMS in their respective positions. There is no agreement on the optimal panel size for a Delphi survey. However, small groups of experts with similar training and understanding of the field of interest have produced reliable results [5]. We selected 20 individuals to provide representative data without generating excess information.

Survey

We used the web-based survey tool Survey Monkey (SurveyMonkey®, available from www.surveymonkey.com) to administer the Delphi survey. Participants initially received an email outlining the purpose and expectations of the study, responding to the survey implied consent. Reminders were sent at two-week intervals twice during each survey round. In order to provide survey reminders and feedback on responses, study authors (AS, MM) knew the identity of respondents.

In round one of the surveys, we provided the participants with a previously developed, comprehensive list of 118 individual emergency ECG diagnoses or findings grouped into six categories: Pacemaker, Ischemia/ST-segment changes, Dysrhythmia/blocks, Genetic, Miscellaneous/ECG findings, Electrolyte/Toxicological [6]. This list was based on a review of the American Board of Internal Medicine 94-question/answer sheet for the ECG portion of the cardiovascular disease board examination and modified using textbooks in emergency medicine (EM) and cardiology as well as review by two EM physicians, each with more than five years of clinical experience. Using the four-point Likert scale and associated descriptors below, the participants individually rated all 118 ECG diagnoses or findings based on the “importance” of paramedic recognition and “impact on prehospital care”: 1) Not necessary, no impact on prehospital care (1 point), e.g. An ECG finding that requires no intervention or change in management in the prehospital phase of care; 2) Minimally important, will have little impact on prehospital care (2 points), e.g. An ECG finding that may require intervention in the ED/hospital but not usually the prehospital environment. Paramedics may monitor the patient closely or draw the attention of ED staff towards the finding but may not have the ability to treat it specifically. Failure of the ACP to recognize the finding is unlikely to harm patients; 3) Important, will have a moderate impact on prehospital care (3 points), e.g., an ECG finding that paramedics can treat and potentially improve the condition the patient. Failure of the ACP to recognize the ECG finding may lead to patient harm. The ECG may also be a vital disease mimic that may lead to improper management and patient harm if misinterpreted; 4) Very important, will have a significant impact on prehospital care (4 points), e.g. An ECG finding that requires urgent or emergent management by the ACP or the patient will experience harm. The ECG may be an essential mimic of disease whereby misinterpretation will lead to inappropriate treatment and patient harm.

A fifth option, “I am not familiar with this diagnosis or finding”, was also available. After each of the first two rounds of the Delphi process, we gave individuals the opportunity to add diagnoses or findings as appropriate. Furthermore, at the end of each round, participants received a summary of their responses and the responses of the group in aggregate (number and proportion of individuals selecting a specific rating). At this time, they could make adjustments to their initial responses if desired. 

Outcomes Measures

We removed diagnoses and findings from subsequent rounds once they reached the primary outcome of consensus or stability. We defined “consensus” as a minimum of 75% agreement among participants on Likert rating for a single diagnosis and/or finding during survey rounds one to three, and “stability” as a shift of 20% or less among participants on individual ratings between rounds (indicating further consideration would be unlikely to bring the group closer to consensus) [6]. Secondary outcomes included a categorization (arbitrarily defined cut-offs) of ECGs and/or findings by final mean Likert rating, differences in both mean Likert ratings and mean standard deviations for ECG and/or finding questions over successive rounds, and differences in mean Likert ratings between healthcare groups (physician/paramedic).

Statistical Analysis

For each ECG diagnosis or finding survey question, each question round and each healthcare professional group, we calculated the mean and standard deviations among the individual Likert ratings (up to 10 ratings per healthcare group). At the same time, we categorized each ECG diagnosis/finding survey question as one of the six ECG categories. We used mixed-effects models to analyze standard deviations and means of Likert ratings for all the questions on successive survey rounds from the two healthcare groups (paramedic, physician) [6]. In order to account for correlations in the outcomes over time with each question, we employed a three-level hierarchical structure in all models, with random effects for questions both overall and within each healthcare professional group. We evaluated the statistical significance of the three-way interaction effect from the factors: ECG category, healthcare group and round. We compared the different residual variance structures of the mixed models. We used an unstructured residual correlation structure in the analyses for both the standard deviations and means of the Likert ratings. If the three-way interaction effect was not statistically significant, we evaluated every two-way interaction effect of the three factors. We included statistically significant interaction terms in the final models.

We based all statistical tests on the Wald test and considered a p-value < 0.05 as statistical significance. Based on residuals, we validated all final models by diagnostic procedures. We performed all statistical analyses using Stata version 16.1 (StataCorp. 2020. Stata Statistical Software: Release 16.1. College Station, TX: StataCorp LLC).

## Results

We recruited 20 participants (ten physicians, ten paramedics). Ninety percent (18) of participants were of male gender (one physician and one paramedic were female genders), and two (20%) of the paramedics identified their primary healthcare role as a paramedic educator. Seventy-five percent (15) of the participants had ten or more years of experience in their current profession, with only one physician (5%) having less than five years of experience. All 20 (100%) completed the first and second rounds, and 17 (85%) completed three rounds. In addition to the original list of 118 diagnoses or findings, participants suggested three findings that we added to the list for a total of 121 ECGs rated (Appendix A).

Overall, 32 (26.4%) of 121 potentially important ECG diagnoses or findings reached consensus (Table 1), two (1.7%) reached stability and 87 (71.9%) reached neither consensus nor stability. Sixteen, five, and 11 ECG diagnoses/findings reached consensus in rounds one, two, and three. When categorized by the final mean Likert rating (Table 2), 77 (63.6%) diagnoses or findings were either “Very Important” (mean rating > 3.7) or “Important” (mean rating 2.7-3.7). A complete list of final mean Likert ratings for all 121 ECG diagnoses or findings can be found in Appendix A.

**Table 1:** Categorization of ECG Diagnoses and/or findings reaching consensus (32/121 total) None of the ECGs in the Miscellaneous/ECG findings category reached consensus or stability; two diagnoses met stability criteria: Left anterior fascicular block, Left posterior fascicular block ECG= electrocardiogram, RBBB= right bundle branch block, LBBB= left bundle branch block, MI= myocardial infarction, AV= atrioventricular, WPW= Wolf Parkinson White

	Very Important (n=21)	Important (n=6)	Minimally Important (n=5)	Not Important (n=0)
Dysrhythmias/blocks	Sinus bradycardia	Sick sinus syndrome	Complete RBBB	
	Supraventricular tachycardia (general)	Sinus tachycardia with WPW	Incomplete RBBB	
	Atrial fibrillation		Incomplete LBBB	
	Wide complex tachycardia (general)			
	Monomorphic ventricular tachycardia			
	Polymorphic ventricular tachycardia			
	Torsades de pointes			
	Multifocal ventricular tachycardia			
	Agonal idioventricular rhythm			
	3^rd^ degree AV block			
Ischemia/ST segment changes	Acute inferior MI		Right ventricular strain pattern	
	Acute lateral MI		Right ventricular hypertrophy	
	Acute anterior MI			
	Acute posterior MI			
	Acute right ventricular MI			
	Acute MI with LBBB (including Sgarbossa’s criteria)			
	Acute MI with RBBB			
	Acute MI in a paced rhythm (including Sgarbossa’s criteria)			
Electrolyte/toxicological disturbances	Hyperkalemia	Calcium channel blocker toxicity		
		Beta blocker toxicity		
Pacemaker	Failure to capture			
	Failure to pace			
Genetic		Brugada syndrome		

**Table 2:** 121 ECG diagnoses/findings by final mean Likert rating ECG= electrocardiogram

	Very Important: Will have significant impact on prehospital care	Important: Will have moderate impact on prehospital care	Minimally Important: Will have little impact on prehospital care	Not Important: Will have no impact on prehospital care
	Mean Rating >3.7	Mean Rating 2.7-3.7	Mean Rating 1.7-2.69	Mean Rating <1.7
Number of ECGs/findings (%)	17 (14)	60 (49.6)	44 (36.4)	0 (0)

**Figure 1:**
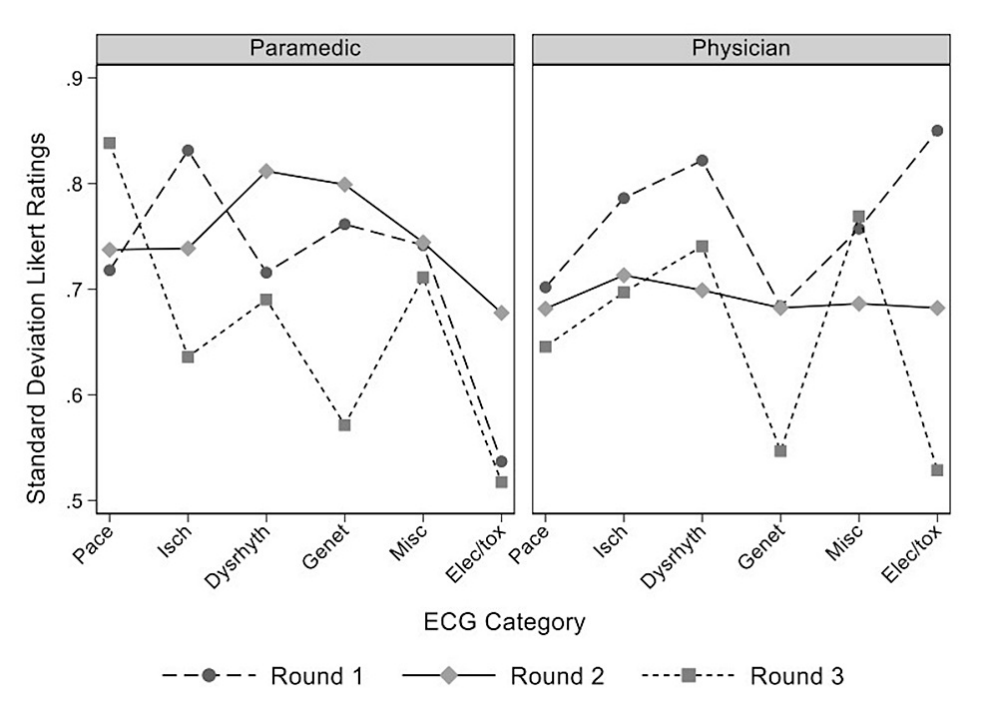
Mean standard deviation of Likert ratings for 95 ECG diagnoses and findings by ECG category and Delphi round Pace= pacemaker, Isch= ischemia/ST segment changes, Dysrhyth= dysrhythmia/blocks, Genet= genetic, Misc= miscellaneous/ECG findings, Elec/tox= electrolyte/toxicological, ECG= electrocardiogram

**Figure 2:**
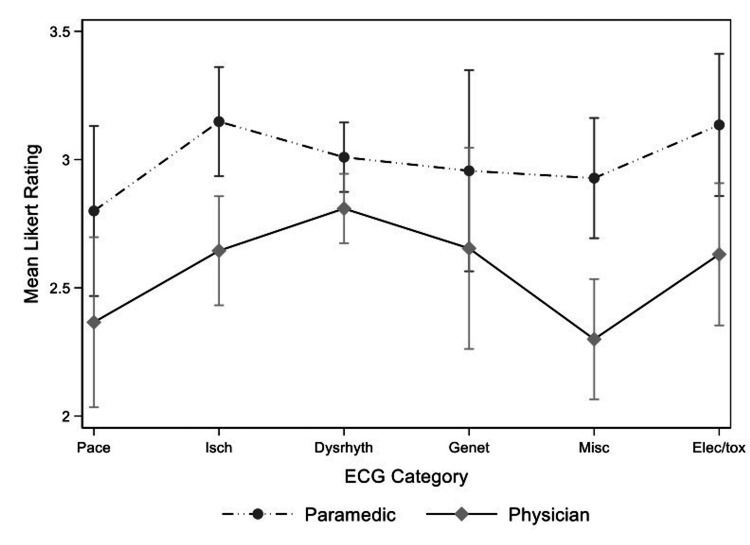
Final mean Likert rating of 95 ECG diagnoses and findings by ECG category and healthcare group Pace= pacemaker, Isch= ischemia/ST segment changes, Dysrhyth= dysrhythmia/blocks, Gen= genetic, Misc= miscellaneous/ECG findings, Elect= electrolyte/toxicological, ECG= electrocardiogram

**Figure 3:**
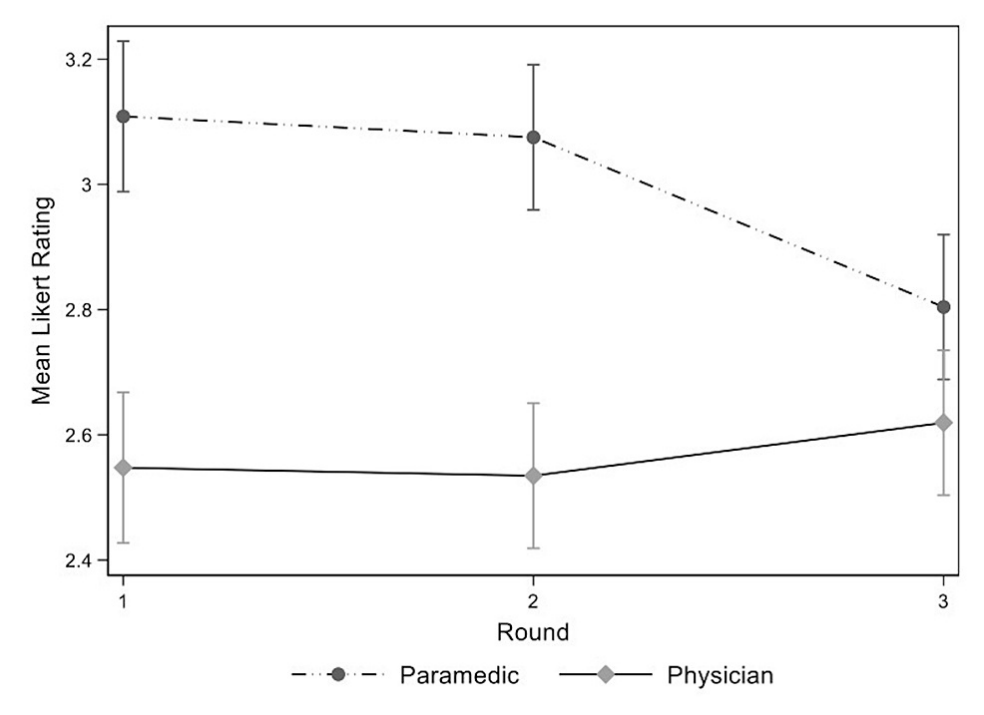
Mean Likert rating of 95 ECG diagnoses and findings by Delphi round and healthcare group ECG= electrocardiogram

We included participants that completed all three rounds of the survey (17, 85%) in the final analysis of both the standard deviations and the predicted means of the Likert ratings. Further, we included 95 ECG diagnoses/findings with responses in all three rounds (i.e. failed to reach consensus or stability in rounds one and two). The three-way interaction effect was significant in the mixed model analysis for the standard deviations of the Likert ratings (p-value = 0.026). Overall, the mean standard deviations of the Likert ratings decreased over successive rounds, but this depended on both the ECG category and healthcare group (Figure 1). In the three-level mixed-effect model analysis for predicted means of the Likert ratings, the three-way interaction effect of ECG category, healthcare group and round was not statistically significant. All two-way interactions of all three factors were significant, and they were included in the final model. Overall, the final predicted means for all ECG categories were lower in the physician group (Figure 2), although only the differences in “Ischemia” and “Miscellaneous” categories were statistically significant. There were statistically significant differences between healthcare groups in round one and two but not in round three (Figure 3).

## Discussion

Our results prioritize emergency ECG diagnoses or findings specifically for prehospital healthcare providers. We believe this list will be helpful to both educational institutions and practising paramedics looking to optimize ECG learning. As expected, critical dysrhythmias (e.g. polymorphic VT) and ischemic/ST-segment changes (e.g. acute anterior MI) most commonly reached consensus and were classified as “Very Important”. Recognition of these diagnoses can significantly and immediately affect patient outcomes. Other items, including third-degree heart block and hyperkalemia, were also rated “Very important”. When evaluating diagnoses/findings by mean Likert rating, approximately 2/3 of diagnoses and findings were rated “Very Important” or “Important”.

A large number (71.9%) of diagnoses/findings failed to reach the defined primary outcome of either consensus or stability. Although the low consensus rate may relate to differences in the subjective interpretation of rating categories (e.g. “Very Important” vs “Important”), it may also suggest that there is existing disagreement on essential ECG knowledge among paramedics or physicians. Importantly, our results demonstrated that over the three rounds, predicted mean and standard deviations decreased in most ECG categories (Figure 1). There was a convergence of mean Likert ratings between healthcare groups (Figure 3). Both of these findings support a move toward consensus among participants. In the first round of the survey, the mean Likert rating of the importance of a paramedic knowing a specific ECG was lower in the physician group compared to the paramedic group on 85 (72%) of 118 initial diagnoses and findings. This trend continued across all three rounds; however, the differences were only statistically significant in two ECG categories (Ischemia/ST-segment changes, Miscellaneous). In the Ischemia category, all ECGs that would potentially meet the criteria for acute reperfusion with prehospital thrombolysis reached consensus in the first two rounds. ECGs remaining for analysis included mostly other acute ischemic ECG changes and ischemia mimics. The clinical management of patients with these findings is often less certain initially and may require specialist cardiology consultation. The additional experience and knowledge of physicians regarding in-hospital care may account for the differences in observed mean ratings between healthcare groups. None of the ECGs in the miscellaneous/ECG findings category reached consensus or stability. Ultimately, the significance of this difference in ratings between paramedics and physicians is uncertain; nevertheless, it is of particular interest and worthy of further exploration given the authority of physicians as medical directors and the current trend towards self-regulation of the paramedic profession.

Past research on paramedic ECG knowledge has primarily focused on the accuracy of diagnosis of Acute Coronary Syndromes (ACS), particularly ST-segment elevation myocardial infarction (STEMI) [7-15]. Overall, there is wide variability in reported paramedic performance in ECG interpretation with sensitivities and specificities for STEMI ranging between 71% and 99% and 53%-96%, respectively [7-13]. A small number of studies have investigated Supraventricular Tachycardia (SVT) and other dysrhythmias [16-18]. The reported rates of paramedic misidentification of SVT range between 3%-31% [16,17], and a single study found the sensitivity for AV block to be as low as 46% [18]. The reasons for variability in performance are unclear; however, studies that included a formal ECG training component of at least eight hours tended to have higher results [7-9]. This is the only study we are aware of that has attempted to prioritize a list of ECG diagnoses for paramedic ECG learning.

Patocka et al. used a similar methodology (including the same ECG list) to investigate what ECGs are vital for trainees in Emergency Medicine (EM). They found consensus among residency program directors that these EM resident physicians “must-know” the majority of ECG diagnoses, similar to cardiologists, and that there is no EM specific “list” that can be used for curriculum development or assessment [6]. In contrast, our study suggests that a focused list may be necessary and valuable in the prehospital context. By focusing ECG learning on critical diagnoses, we predict that paramedics may improve diagnostic sensitivity and specificity. Nevertheless, we do not advocate for the replacement of basic ECG interpretation skills with rote memorization alone. Clinical practice requires recognizing the ECG findings and applying the knowledge in the context of patient care. Given that students are unlikely to experience clinical examples of every vital ECG finding on their practical rotations, a list of essential diagnoses may supplement and focus learning.

Study Strengths and Limitations

Study strengths included using a standardized list of diagnoses or findings and recruiting paramedics and physicians from all Canadian provinces. Although open discussion may have led to a higher consensus, we conducted our survey in a quasi-anonymous fashion to avoid the possibility of influence through group dynamics. Arguably, the physician and paramedic groups are sufficiently heterogeneous that they should not be combined in this type of study. Given each group’s prominence in prehospital medicine, we did not feel it would be possible to conduct this study without engaging both. Finally, the small panel size may have limited the generalizability of responses, but a larger group may have also resulted in participant fatigue with the increased amount of data generated.

## Conclusions

We have created a list of ECG diagnoses or findings prioritized for the EMS context. Educators may use this list to focus on interventions aimed at ECG teaching. Further research should examine potential differences between paramedics and physicians concerning what essential medical knowledge is required for clinical practice.

## Appendices

Final mean rating for 121 ECG findings and or diagnoses 

Present in round 3

Oversensing: 2.47

Undersensing: 2.76

Pacemaker Mediated Tachycardia: 2.71

Runaway Pacemaker: inappropriately rapid discharges: 2.88

Atrial Paced Rhythm: 2.12

Dual-chamber Paced Rhythm: 2.18

Right Ventricular Paced rhythm: 2.12

Left Ventricular Aneurysm: 2.29

Left Ventricular Hypertrophy: 2.47

Left Ventricular Strain Pattern: 2.41

Right Ventricular Hypertrophy: 2

Benign Early Repolarization: 3.12

Prinzmetal's Angina: 2.82

Central Nervous System Injury: 2.41

Changes of Chronic Myocardial Infarction: 3

Wellens Syndrome: angina with T-wave inversion or biphasic T-waves in the left anterior descending territory: 3.29

Pericarditis: 3.18

Hypokalemia: 2.47

Hypercalcemia: 2.24

Hypocalcemia: 2.29

Digitalis Toxicity: 2.65

Digitalis Effect: 2.18

Beta Blocker Toxicity: 3.06

Calcium Channel Blocker Toxicity: 3.06

Sodium Channel Blockade Toxicity: 3.29

Cocaine Intoxication: 3.12

Hypothermia: 3.12

Athlete's Heart:  physiologic adaptations to chronic intensive exercise: 2.06

Dextrocardia: 2.18

Incomplete Sinoatrial Block:  occasionally blocked P waves: 2.42

Complete Sinoatrial Block:  sustained failure of the sinus node to pace the heart: 3.29

Sick Sinus Syndrome:  group of dysrhythmias with the hallmarks of alternating bradycardia and tachycardia: 2.94

1st Degree Atrioventricular Block: 2.18

2nd Degree Atrioventricular Block Mobitz Type 1 Wenckebach: 3

2nd Degree Atrioventricular Block Mobitz Type 2: 3.59

Junctional Escape Rhythm: 3.41

Wandering Atrial Pacemaker: 2.47

Sinus Tachycardia: 3.53

Inappropriate Sinus Tachycardia:  condition without apparent heart disease or other causes of sinus tachycardia: 3.06

Sinus Tachycardia with Bundle Branch Block: 3

Sinus Tachycardia with Wolf Parkinson White: 3

Supraventricular Tachycardia specific diagnosis Sinoatrial Nodal Reentrant Tachycardia: rare tachycardia characterized by an atrial rate between 105-150 beats per minute and P-wave morphology similar to sinus rhythm: 2.76

Supraventricular Tachycardia specific diagnosis of Atrial Tachycardia: 3.06

Supraventricular Tachycardia specific diagnosis of Junctional Tachycardia:  any tachycardia that involves the atrioventricular node: 2.82

Supraventricular Tachycardia specific diagnosis of Atrioventricular Nodal Reentrant Tachycardia:  a form of junctional tachycardia where the reentrant circuit is confined to the atrioventricular node: 2.82

Supraventricular Tachycardia specific diagnosis of Atrioventricular Reciprocating Tachycardia:  a form of junctional tachycardia where the reentrant circuit involved the atrioventricular node as well as an accessory pathway: 2.82

Supraventricular Tachycardia specific diagnosis of Accelerated Junctional Tachycardia aka nonparoxysmal junctional tachycardia:  focal sites of enhanced automaticity/triggered activity in the atrioventricular node or bundle of His: 2.71

Supraventricular Tachycardia specific diagnosis of Paroxysmal Junctional Tachycardia aka focal junctional tachycardia:  primarily occurs in children, occurs at rates up to 250 beats per minute and can be irregular: 2.76

Atrial Fibrillation with Slow Ventricular Escape Rhythm: 3

Atrial Fibrillation with Bundle Branch Block: 2.94

Atrial Fibrillation with Pre-Excitation (e.g. Atrial fibrillation with Wolf Parkinson White): 3.41

Atrial Flutter: 3.41

Atrial Flutter with Slow Ventricular Escape Rhythm: 3.06

Atrial Flutter with 2:1 Conduction: 2.94

Atrial Flutter with 3:1 Conduction: 2.88

Atrial Flutter with Variable Conduction: 2.88

Multifocal Atrial tachycardia (MAT): 2.53

Ventricular Escape Rhythm: 3.59

Agonal Idioventricular Rhythm: 3.59

Accelerated Idioventricular Rhythm: 3.41

Bidirectional Ventricular Tachycardia: 3.12

Brugada Syndrome: 2.76

Long QT Syndrome: 3.35

Hypertrophic Cardiomyopathy: 2.47

Wolff-Parkinson White Syndrome: 3.24

Arrhythmogenic Right Ventricular Dysplasia:  genetic cardiomyopathy characterized by ventricular arrhythmias (findings include T wave inversions, right bundle branch block pattern and epsilon waves): 1.88

Incomplete Right Bundle Branch Block: 1.76

Complete Right Bundle Branch Block: 2.12

Incomplete Left Bundle Branch Block: 1.94

Complete Left Bundle Branch Block: 3.06

Rate Related Bundle Branch Block: 2

Bifascicular Block: 2.06

Nonspecific Intraventricular Conduction Delay: 1.71

ST elevation in aVR: 3.65

PR depression in aVR: 2.29

+30 degrees R-wave axis in aVR: 1.71

Early Reciprocal Change in aVL: 3

Reciprocal Changes: 3.59

S1Q3T3: 2.06

Morphologic Patterns Seen with Pulmonary Embolism: 2.53

Low Voltage: 2.18

Artifact: 3.41

Precordial Lead Misplacement: 3.29

Limb Lead Misplacement: 3.29

Post-Electrical Cardioversion Changes: 2.76

Post-tachycardia T-wave Pattern: 2.12

Prominent T-waves: 3

Inverted T-waves: 3.24

Biphasic T-waves :2.89

Persistent Juvenile T-Wave Pattern: 1.76

Normal Axis: 2.29

Abnormal Axis: 2.35

Premature Atrial Contraction: 2.35

Premature Junctional Contraction: 2.35

Premature Ventricular Contraction: 2.53

P wave changes with Chronic Obstructive Pulmonary Disease: 1.82

Left Main Coronary Artery Occlusion (wide spread horizontal ST depressions most prominent in leads I, II and V4-6, ST elevated in aVR > 1mm, ST elevation in aVR ≥ V1): 3.47

De Winter’s T Waves (an anterior ST-elevation myocardial infarction equivalent) key features include ST depression and peaked T waves in the precordial leads: 3.12

Removed after round 1

Failure to Pace: 3.75

Failure to Capture: 3.75

Acute Inferior Myocardial Infarction: 4

Acute Lateral Myocardial Infarction: 3.9

Acute Anterior Myocardial Infarction: 3.95

Acute Posterior Myocardial Infarction: 3.95

Acute Right Ventricular Myocardial Infarction: 4

Hyperkalemia: 3.8

Sinus Bradycardia: 3.6

3rd Degree Atrioventricular Block: 3.95

Supraventricular Tachycardia: general recognition of a narrow complex tachycardia originating above the ventricles: 3.9

Atrial Fibrillation: 3.7

Wide Complex Tachycardia: general recognition of a wide complex tachycardia originating below the atria: 3.95

Monomorphic Ventricular Tachycardia: 3.85

Polymorphic Ventricular Tachycardia: 3.9

Torsades de Pointes: 3.8

Removed after round 2

Acute Myocardial Infarction with Right Bundle Branch Block: 3.75

Acute Myocardial Infarction with Left Bundle Branch Block (including use of Sgarbossa's criteria): 3.8

Acute Myocardial Infarction in a Paced Rhythm (including use of Sgarbossa's criteria): 3.8

Right Ventricular Strain Pattern: 2.1

Multifocal Ventricular Tachycardia: 3.6

Left Anterior Fascicular Block: 1.8

Left Posterior Fascicular Block: 1.8
